# Low-Pressure Burst-Mode Focused Ultrasound Wave Reconstruction and Mapping for Blood-Brain Barrier Opening: A Preclinical Examination

**DOI:** 10.1038/srep27939

**Published:** 2016-06-13

**Authors:** Jingjing Xia, Po-Hsiang Tsui, Hao-Li Liu

**Affiliations:** 1 Department of Electrical Engineering, Chang Gung University, Taoyuan, Taiwan; 2 Institute of Biomedical and Health Engineering, Shenzhen Institute of Advanced Technology, Chinese Academy of Sciences, Shenzhen, China; 3 Medical Imaging Research Center, Institute for Radiological Research, Chang Gung University and Chang Gung Memorial Hospital at Linkou, Taoyuan, Taiwan; 4 Department of Medical Imaging and Radiological Sciences, Chang Gung University, Taoyuan, Taiwan

## Abstract

Burst-mode focused ultrasound (FUS) exposure has been shown to induce transient blood-brain barrier (BBB) opening for potential CNS drug delivery. FUS-BBB opening requires imaging guidance during the intervention, yet current imaging technology only enables postoperative outcome confirmation. In this study, we propose an approach to visualize short-burst low-pressure focal beam distribution that allows to be applied in FUS-BBB opening intervention on small animals. A backscattered acoustic-wave reconstruction method based on synchronization among focused ultrasound emission, diagnostic ultrasound receiving and passively beamformed processing were developed. We observed that focal beam could be successfully visualized for *in vitro* FUS exposure with 0.5–2 MHz without involvement of microbubbles. The detectable level of FUS exposure was 0.467 MPa in pressure and 0.05 ms in burst length. The signal intensity (SI) of the reconstructions was linearly correlated with the FUS exposure level both *in-vitro* (r^2^ = 0.9878) and *in-vivo* (r^2^ = 0.9943), and SI level of the reconstructed focal beam also correlated with the success and level of BBB-opening. The proposed approach provides a feasible way to perform real-time and closed-loop control of FUS-based brain drug delivery.

In the central nervous system (CNS), the blood–brain barrier (BBB) prevents larger molecules (>400 Da) from entering the brain parenchyma, protecting it from toxic foreign substances^1^,^2^. Recently, it was discovered that the presence of microbubbles combined with low-energy burst-tone focused ultrasound (FUS) exposure can produce local and temporary disruption of BBB^3^,^4^. This noninvasive procedure temporally disrupts the BBB locally rather than systemically, minimizing off-target effects. It provides a window of opportunity to achieve local delivery of therapeutic agents into the brain with either an intact or compromised BBB.

When delivering drugs to the brain, the FUS energy needs to be guided with high precision during intervention. Currently, contrast-enhanced MRI is the most reliable approach to monitor the occurrence of BBB opening postoperatively by intravenously administering the MR contrast agent postoperatively and detecting the imaging signal intensity change caused by the contrast agent local leakage^5^,^6^,^7^. However, BBB opening can only be confirmed post-operatively. MRI previously served as an excellent guidance tool for thermal therapy application since intense and continuous-wave (CW) FUS induces temperature change to cause linear proton-resonance-frequency (PRF) shift and enable temperature-dependent magnitude/phase change in MRI^8^. Yet, guidance of non-thermal applications such as FUS-induced BBB opening is challenging since MRI fails to detect burst-tone weak-pressure ultrasound beam pattern exposure. Recent novel advance such as MR-based acoustic-radiation-force imaging (ARFI) still requires CW mode exposure with an acoustic pressure of 4–6 MPa to detect acoustic patterns^9^,^10^, whereas much higher than the required pressure level of sub-MPa level in FUS-BBB opening application. Therefore, there is still strong demand to develop means to identify FUS patterns under a burst-mode weak exposure for real-time guidance of FUS-BBB opening.

Another potential alternative approach is passively beamformed analysis. Previously, researchers investigated the use of backscattered acoustic-wave detection to characterize high-pressure CW ultrasound exposure, since intense ultrasound exposure typically accompanies temperature-induced boiling or acoustic cavitations to produce strong backscattered acoustic waves^11^,^12^,^13^. Diagnostic ultrasound probe was also recently been attempted to collect multiple-channel backscattered acoustic waves to construct emitted focal beam patterns. This approach uses a passive conventional algorithm based on frequency-14,15 or time-domain^16^ passive beamformed theory applied in diagnostic ultrasound imaging which were originally developed for seismic imaging^17^,^18^,^19^,^20^. Haritonova *et al*. reported a focal-beam pattern visualization development on a thermal-therapy purposed dual-mode therapy array can successfully visualize the continues-mode beam pattern down to 1.732 MPa^21^. Lately, passive imaging has been of high interest in ultrasound research due to its potential to map bubble activity during cavitation-enhanced therapies, thereby enhancing treatment safety and assessing outcome^16^,^22^,^23^,^24^,^25^,^26^. Passive imaging approaches have been shown to track individual sites of cavitation activity with sub-millimeter spatial and millisecond temporal accuracy^27^ and have been used to effectively detect inertial cavitation events and predict high-intensity FUS-induced lesion formation (exposure level of 1.15- 2.50 MPa can be detected)^22^, and the monitoring of microbubble-seeded cavitation dynamics^15^,^17^. So far approach based on passive beamforming approach with the involvement of microbubbles can detect ultrasound patterns down to sub-MPa level^28^,^29^. However, none of studies so far have shown successful focal beam reconstruction with low-pressure burst-tone mode exposure condition without involvement of microbubbles. The presence of microbubbles in FUS exposure already induces capillary effect, and would be challenging when intending to decouple focal beam guidance/assurance and the eventual BBB-opened intervention.

In this study, we first time proposed a novel approach using backscattered acoustic wave construction to achieve: (1) *in-vitro* visualization of burst-tone weak-pressure focused ultrasound pattern without microbubbles, and (2) *in-vivo* exposure-level estimation to perform focal beam guidance for FUS-induced BBB-opening assurance. We developed a synchronized algorithm to reconstruct the acoustic wave maps, and tested the beam pattern reconstruction capabilities under different exposure frequencies, exposure burst lengths, acoustic pressures and frame numbers. We also demonstrated that the reconstructed acoustic wave pattern may be utilized to provide imaging feedback to guide the ultrasonic emission-related therapy.

## Results

### Backscattered wave reconstruction allows short-burst low-pressure focused ultrasound visualization

**Figure 1:**
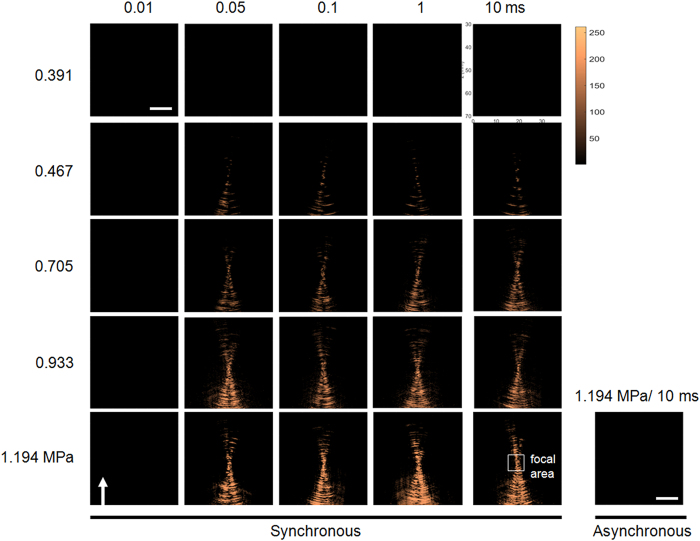
A summary shows acoustic wave reconstruction maps of FUS exposures with different exposure burst lengths (0.01–10 ms) and different exposure levels (0.391–1.194 MPa). The bottom-right subplot shows asynchronous implementation of reconstruction under the exposure of 1.194 MPa and 10-ms burst length. Arrows indicate the FUS emit direction.

First, we investigated the effect of exposure level by testing the acoustic wave reconstruction at different exposure levels with a default tested transducer frequency of 1.5 MHz (theoretical deviations of the proposed backscattered acoustic-wave reconstruction are shown in the Supplementary method). To show the proposed approach was suitable for capturing short-period ultrasound exposure, we compared the acoustic wave reconstructed maps of ultrasound exposures with various burst length. Figure 1 shows an experiment using a 1.5-MHz focused ultrasound transducer to deliver excitation with the pressure ranging from 0.391 to 1.194 MPa and a burst length ranging from 0.01-ms to 10-ms. On one hand, the wave front can be identified when the pressure value exceeded 0.467 MPa, but not when the pressure was lower than 0.391 MPa. Further, the signal level of the reconstructed maps seemed to be increased with increasing pressure levels. Besides, this experiment also showed that the reconstructed maps from 0.05-ms to 10-ms excitation can identify the wave front of the propagated ultrasonic waves, but the wave fronts cannot be identified for excitation lengths equal to or less than 0.01-ms. In summary, the map quality was not obviously effected by the exposure burst length, but was dominated by exposure level. For comparison, we also showed that it was failed to reconstruct the focal beam patterns with the asynchronous data (with exposure condition of 1.194 MPa and 10 ms of burst length), indicating the necessity of synchronization between FUS exposure and backscattered acoustic wave receiving for short-burst ultrasound beam visualization (Fig. 1; bottom-right subplot).

**Figure 2:**
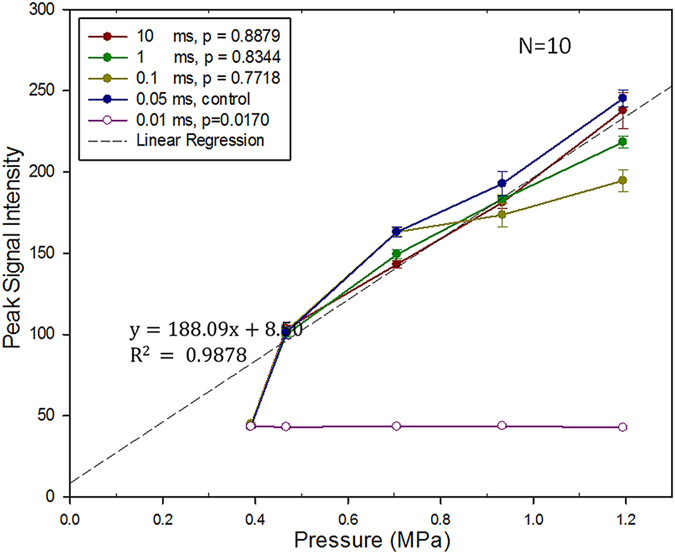
Peak signal intensity of the acoustic wave reconstruction maps under different exposure levels (0.391–1.194 MPa) and different burst lengths (0.01–10 ms; frequency = 1.5 MHz). The dashed line represents the linear regression when pressure >0.467 MPa and burst length >0.05 ms. N = 10 for each exposure condition. The *p* values was calculated when using 0.05-ms burst length group as control.

The relationships between the peak signal intensity (SI) of the estimated beam patterns with various exposure burst lengths as well as exposure pressure levels were also investigated (Fig. 2). We defined SI as the average of the square root of the data acquired from ROI (size: 1.5 mm × 1.5 mm) covering beam pattern. Beam patterns were detectable for all tested pressures under the burst length reaching 0.05 ms. As exposure level increased to greater than 0.467 MPa, the peak SI showed a linear correlation with the increased exposure level, but was nearly independent to burst lengths (The *p* values among 0.05- to 10-ms exposures were all >0.05). When excluding those failed low-exposure cases, there was a high linear regression (y = 138.09x + 8.50, R^2^ = 0.9878), indicating that the reconstruction map intensity was highly correlated with exposure level.

**Figure 3:**
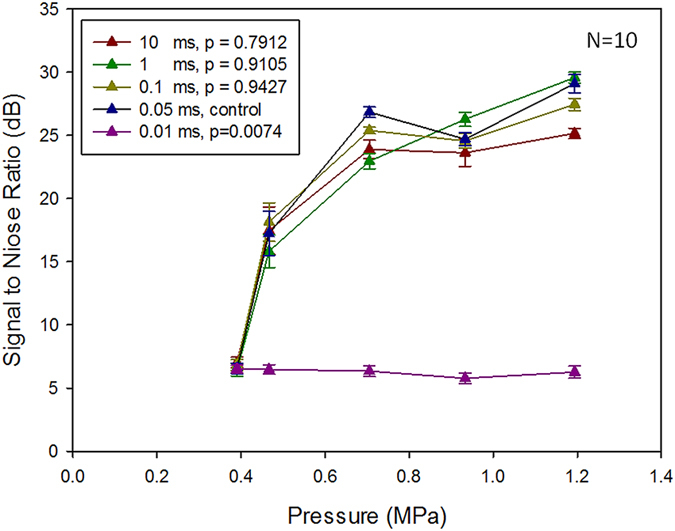
Signal-to-noise ratio (SNR) of acoustic wave reconstruction maps under different exposure levels (0.391–1.194 MPa) and different burst lengths (0.01–10 ms; frequency = 1.5 MHz). N = 10 for each exposure condition. The *p* values was calculated when using 0.05-ms burst length group as control.

Figure 3 shows the corresponding signal-to-noise ratio (SNR) of acoustic wave reconstruction maps. SNR level was 16 ± 4.75 dB at 0.467-MPa exposure (for all detectable burst length), and increased to 23.48 ± 7.49 dB at 0.705-MPa exposure (for all detectable burst length) and then remained plateau as the pressure further increased. Statistical analysis showed no significant difference in SNR change among various detectable burst-length groups, indicating that SNR level was nearly independent to the burst length when exposure pressure reaching 0.705 MPa. SNR seemed to increase as the exposure level increased, but not as the exposure burst length. Together with Figs 2 and 3 illustrated that both the reconstructed acoustic wave level and the acoustic wave reconstruction map quality depend heavily on the ultrasound exposure level, implying that the peak SI level of the reconstructed beam patterns accurately reflects the exposure pressure in *in vitro* phantoms.

**Figure 4:**
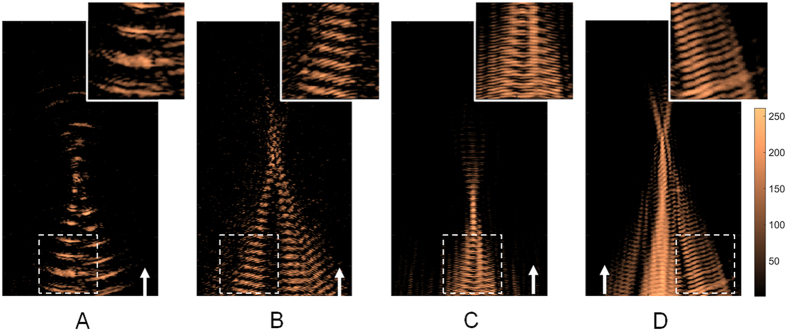
Acoustic wave reconstruction maps of FUS exposures with different exposure frequencies. (**A**) 0.55 MHz, 0.8 MPa; (**B**) 1.1 MHz, 0.8 MPa; (**C**) 1.5 MHz, 0.9 MPa; (**D**) 2 MHz, 1.1 MPa. In all experiments the burst length were set to 10 ms. N = 10 for each exposure condition. Arrows indicate FUS exposure direction.

Figure 4 shows representative examples of using the proposed approach to reconstruct the focal beam patterns from a 10-ms exposure. We demonstrated that the focal beam pattern delivered from a 0.55 to 2 MHz FUS transducer can be successfully reconstructed. These results showed that the proposed algorithm is capable of visualizing the acoustic maps with single short-burst FUS exposure. It was noted that the algorithm successfully reconstruct the acoustic emission maps although the exposure signals (locates at 0.55–2 MHz) did not well match the receiving probe been employed (i.e., 5–10 MHz).

### Short-burst FUS wave can be captured and reconstructed using single-frame backscattered wave reconstruction

**Figure 5:**
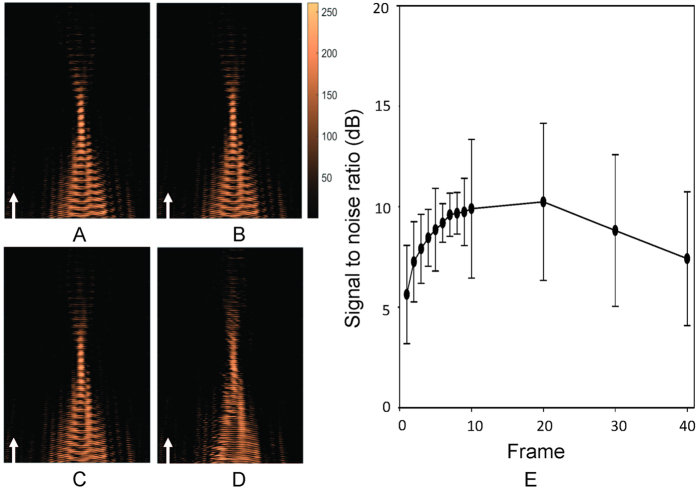
Influence of the frame averaging on focal beam visualization. (**A**) Acoustic wave reconstruction maps of focused ultrasound exposures (1.5 MHz, 0.9 MPa, 10 ms) with different numbers of averaged frames (frame number = 1, 5, 10, 20); (**B**) Signal-to-noise ratio (SNR) of acoustic wave reconstruction maps under different numbers of averaged frames. Arrows indicate FUS exposure direction.

The above results (Figs 2 and 4) demonstrated that the proposed acoustic wave construction algorithm can successfully provide FUS beam patterns with low-pressure short-burst exposure. Next, we investigated the effect of construction frame-averaging. Figure 5(A) provides typical examples of a reconstructed acoustic wave map conducted with different frame-averaging numbers (1, 5, 10 and 20 captures of single 10-ms bursts of FUS excitation in (A)-(D), respectively). The reconstructed beam patterns could be identified under different amounts of frame-averaging; yet, the wave front could be clearly identified in the low frame-averaging setting, but the time errors at each synchronization time point began to accumulate and wave front synchronization between frames gradually lost alignment and moving averaging effect was introduced. The SNR corresponding to the number of averaged frames is shown in Fig. 5(B). The acoustic wave maps showed a 5-dB SNR improvement with the number of averaged frames reached 20, but the SNR level were saturated or even decreased when average number of frames was increased further. The SNR increase due to frame averaging did not present a monotonic increase as the frame number increased, implying that even a single map reconstruction still provides good SNR, and maximally a <5-dB SNR loss when compared with the optimized frame-averaging case. The above supported the capability of capturing short-burst FUS waves via using single-frame capture from the proposed algorithm and system setup.

### Preclinical *in-vivo* experiments support the feasibility to guide ultrasound-induced blood-brain barrier opening

Next, we aimed to employ the proposed acoustic wave reconstruction algorithm to identify focal beam patterns and to test the potential use in guiding the FUS-BBB opening procedure on small animals. Figure S3 illustrates the concept to co-localize ultrasound B-mode imaging, backscattered acoustic emission reconstruction mapping and MRI together to perform FUS-BBB opening. While the a-prior obtained MRI provides a high-resolution anatomical reference, ultrasound imaging provides a high temporal resolution anatomical position orientation to allow coordination of the reconstructed focal beam pattern distribution.

**Figure 6:**
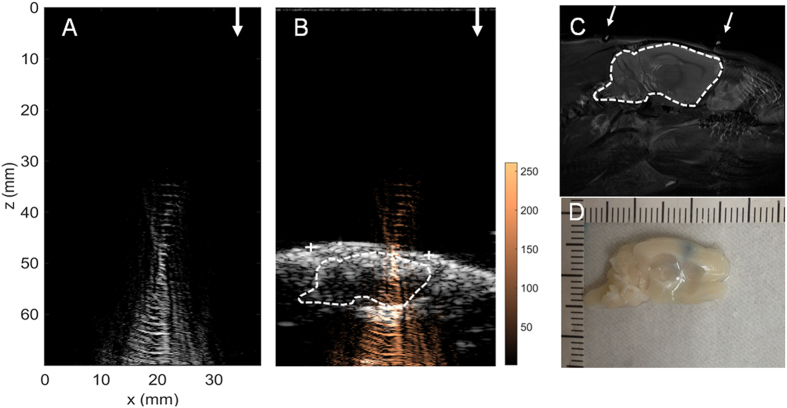
*In vivo* FUS-exposure animal treatment example 1. This example shows the use of intermediate FUS exposure level to perform FUS-BBB opening (frequency = 1.5 MHz, acoustic power = 4.54 W, acoustic pressure = 0.467 MPa). (**A**) Acoustic wave reconstruction maps of FUS exposures (FUS exposure directions are pointed out by arrows); (**B**) Acoustic wave reconstruction maps co-localized with diagnostic ultrasound and MRI (fiducial marker positions are marked as “+”); (**C**) MR images as high-resolution anatomical reference; (**D**) EB-Stained brain section. Arrow heads indicate the attached fiducial markers.

Figure 6 describes the *in vivo* treatment showing the use of intermediate FUS exposure level to perform FUS-BBB opening (frequency = 1.5 MHz, acoustic power = 4.54 W, acoustic pressure = 0.467 MPa after skull penetration; rat skull causes about 10% of pressure loss). The focal beam maps can be easily generated and monitored, and with the co-localization of diagnostic ultrasound and MRI, the focal beam deposition can be clearly identified (peak SI = 168.41). The BBB-opening was successfully induced (confirmed in brain sections), and the BBB-opened positions correlated well with the geometric center of the reconstructed focal beam (compare Fig. 6B,D), indicating that the reconstructed maps provide effective FUS energy prediction during the treatment.

**Figure 7:**
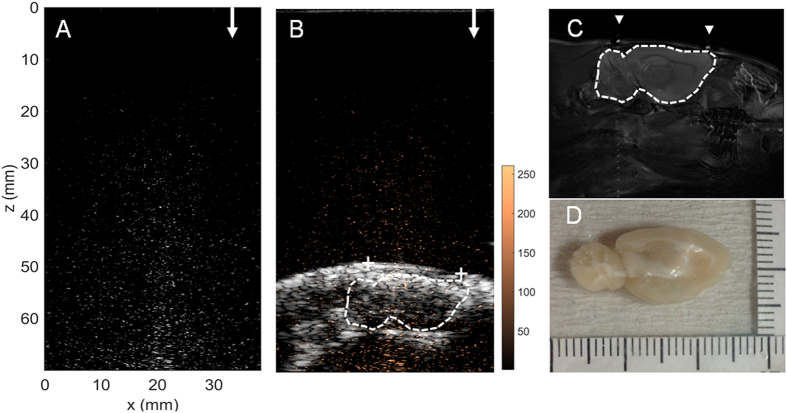
*In vivo* FUS-exposure animal treatment example 2. This example shows the use of intermediate FUS exposure level to perform FUS-BBB opening, but intentionally left strong interference on animal scalp (frequency = 1.5 MHz, acoustic power = 4.54 W, acoustic pressure = 0.467 MPa). (**A**) Acoustic wave reconstruction maps of FUS exposures (FUS exposure directions are pointed out by arrows); (**B**) Acoustic wave reconstruction maps co-localized with diagnostic ultrasound and MRI (fiducial marker positions are marked as “+”); (**C**) MR images as high-resolution anatomical reference; (**D**) EB-Stained brain section. Arrow heads indicate the attached fiducial markers.

In comparison with the first example in Fig. 6, in a second round of experiments (Fig. 7), we employed an identical exposure level (i.e., 0.467 MPa) with the previous case, but intentionally introduce imperfect wave coupling by not perfectly conducting fur shaving of animal scalp. In this case, the BBB-opening was not successfully induced as brain sections showed no EB dye leakage or staining (Fig. 7D), and the focal beam cannot be reconstructed by co-localizing acoustic wave reconstruction maps with MRI and B-mode ultrasound imaging (Fig. 7A,B). The failure of treatment was successfully reflected by the failure of focal beam reconstruction obtained during the exposure (peak SI = 61.16).

**Figure 8:**
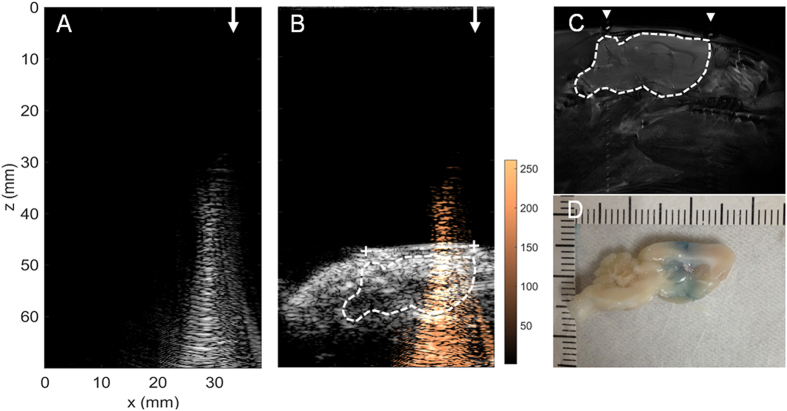
*In vivo* FUS-exposure animal treatment example 3. This example shows the use of high FUS exposure level to perform FUS-BBB opening (frequency = 1.5 MHz, acoustic power = 9.12 W, acoustic pressure = 0.705 MPa). (**A**) Acoustic wave reconstruction maps of FUS exposures (FUS exposure directions are pointed out by arrows); (**B**) Acoustic wave reconstruction maps co-localized with diagnostic ultrasound and MRI (fiducial marker positions are marked as “+”); (**C**) MR images as high-resolution anatomical reference; (**D**) EB-Stained brain slice. Arrow heads indicate the attached fiducial markers.

In the third animal treatment example (Fig. 8), the FUS exposure level was increased for comparison with former *in vivo* experiments (frequency = 1.5 MHz, acoustic power = 9.12 W, acoustic pressure = 0.705 MPa). A reconstructed focal beam pattern with an increased SI signal (peak SI = 271.14) obtained during exposure was observed. The increase of the peak SI level was observed to be ~160% higher than the first treatment case (compare Fig. 8A with Fig. 6A), which corresponds well with a MI increase (~150% higher than the first treatment case), implying that SI change in the reconstruction maps can well reflect the exposure level in the animal brain and outcome of the treatment. Histological examination also confirmed the successful treatment, which was more profound than that with the 0.467-MPa exposure (compare Fig. 8D with Fig. 6D). In 0.705-MPa exposure (group D), erythrocyte extravasations was also observed accompanying with BBB opening, while the extravasations was not observed in the 0.467-MPa exposure (Group C; the corresponding HE stains are shown in Fig. S4). The BBB-opened regions matched with the co-localized MRI/ultrasound/beam mapping imaging and the peak SI showed a good correspondence to the exposure energy level (compare Fig. 8B,D).

**Figure 9:**
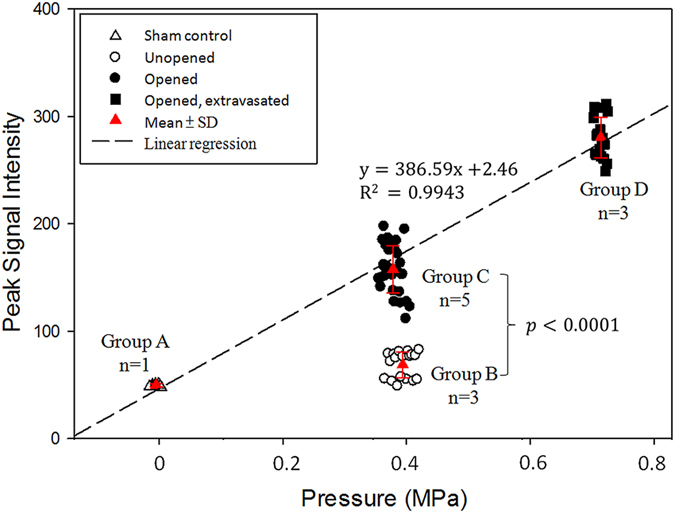
Correlation of the peak SI level with the exposure level obtained from *in vivo* experiments. Four groups of different FUS-BBB sonication levels and FUS-BBB opening outcomes: (Group A): low-level exposure, pressure = 0.111 ± 0.0045 MPa, BBB intact; (Group B): intermediate-level exposure with strong scalp interference, pressure = 0.439 ± 0.014 MPa, BBB intact; (Group C): intermediate-level exposure, pressure = 0.4352 ± 0.0162 MPa, BBB opened; (Group D): high-level exposure, pressure = 0.705 ± 0.005 MPa, BBB opened.

Figure 9 summarized the dependence of the exposure level with the peak SI level during exposure. The experiments were divided into four groups based on the FUS-BBB sonication levels and FUS-BBB opening outcome: low-level exposure (Group A; pressure = 0.11 ± 0.01 MPa, corresponding SI = 49.69 ± 1.16), intermediate-level exposure with strong scalp interference (Group B; pressure = 0.44 ± 0.01 MPa, corresponding SI = 69.53 ± 11.99), intermediate-level exposure (Group C; pressure = 0.42 ± 0.01 MPa, corresponding SI = 160.20 ± 16.14) and high-level exposure (Group D; pressure = 0.703 ± 0.004 MPa, corresponding SI = 279.42 ± 15.59). Besides of group B, the acoustic wave level of groups A, C, and D was observed to be linearly correlated with the acoustic exposure level (y = 386.59x + 2.46, r^2^ = 0.9943) and with signal intensity all statistically distinguishable among groups (all *p* < 0.0001), implying that acoustic wave SI level can effectively serve as an indicator to estimate the exposure level deposited into the brain. When comparing the group C with group B (identical exposure level but C/B are FUS energy with perfectly/poorly penetrated), the acoustic wave SI level provide high distinguished detectability (*p* < 0.0001) to identify the success/failure focal beam energy penetration. This implies that the acoustic wave reconstruction map provide effective index to well consider focal beam penetration, diffraction, and attenuation effects during the exposure. Putting these *in vivo* experimental tests together, it may be feasible to use backscattered acoustic wave level to predict the exact ultrasound deposition in rat brains, therefore may provide a strategy to conduct feedback control for FUS exposure fine adjustment and may have potential to be applied for guidance during FUS-BBB opening on rat brain.

## Discussion

In this study, we investigated a synchronized backscattered acoustic-wave reconstruction approach in order to visualize short-burst low-pressure focused ultrasound beams. By implementing this approach in a commercialized diagnostic ultrasound system, the focal beam patterns can be successfully visualized with a wide FUS exposure frequency range that typically been employed for BBB-opening application (0.5–2 MHz). To our knowledge, the proposed scheme is the first reconstruction method that enables focal beam pattern visualization with a burst length down to 0.01 ms and exposure level down to 0.39 MPa (equivalents to MI of 0.3), with the imaging process without the involvement of microbubbles or acoustic cavitations (it should be noted that current threshold to open the BBB in microbubble-presented FUS exposure is about 1–10 ms of burst length with MI level reaching 0.46^30^). We also proposed to co-localize the focal beam visualization with diagnostic ultrasound imaging as well as pre-acquired MRI, so that the focal beam can be successfully guided to a specific brain target and perform precise FUS exposure control during the intervention. The proposed approach will be valuable when attempting to perform FUS beam pattern visualization and monitoring, and can be beneficiary when using the proposed approach for FUS exposure guidance for future CNS drug delivery application.

Previously, intense (typically 5–10 MPa in pressure in CW excitation) and continuous-wave (CW) FUS energy exposure were typically applied for thermal ablation purpose to induce sufficient energy/viscous heating accumulation (acoustic cavitation is typically involved during this process). It was shown that the reconstruction of backscattered acoustic waves enabled the identification of CW-mode FUS exposure, but the approach still required bubble cloud generation as a strong backscattered source^13^,^31^. Since a bubble cloud can only be generated when acoustic cavitation occurs, intense pressure from the therapeutic ultrasound is required. More recently, a number of reports have introduced microbubbles as an effective cavitation nuclei substitute, and therefore the ultrasound exposure level can be significantly reduced to a sub-MPa level similar to reported level reported from this study^13^,^28^,^31^. It may also possible to employ the non-synchronized reconstructed strategy to perform beam pattern visualization and then concurrently to implement real-time BBB-opening control^28^. However, it should be noted that, with the administration of microbubbles, FUS exposure under such exposure level concurrently induces capillary effects at the targeted area. The synchronization-based reconstruction strategy therefore is advantageous in providing extra flexibility to allow focal beam pattern visualization for pre-treatment evaluation and without inducing CNS capillary effect (since no microbubbles are presented).

For traditional beamformed engines designed for diagnostic ultrasound imaging, B-mode imaging is typically reconstructed based on the collection via a series of A-line acquisitions (64 or 128), thus requires transmission and reception processing times to intrinsically hamper the implementation of short-burst FUS exposure detection (for example, frame rate of 50–100 Hz in B-mode imaging equates to the required time slot of 10–20 ms). In this study, a diagnostic ultrasound engine with beamforming was designed and implemented in a broad-beam “zone”-like plane-wave approach that can complete the single B-mode frame in an extremely short period. These zones are much broader than conventional line-per-line acquisitions and this method typically increases the frame rate by more than 100-fold (completes a frame in tens of microseconds, and the B-mode frame rate is up to 1 kHz)^32^, which makes it possible to track transient activities of short-burst ultrasonic beam propagations. Moreover, synchronization between FUS and the plane wave-based beamforming allows the capturing of backscattered emission events from short-burst FUS exposures. Another advantage is, traditional asynchronization approach can detect cavitation event only when FR reaches 400 frames/s^31^ and in our synchronization method, beam patterns can be reconstructed down to a very low data-capture dynamics (2 frames/s). This not only raises data acquisition efficiency but also reduces workload of the machine memory. Bridging the concepts of ultrafast plane wave-imaging and synchronized acquisition, we anticipated that only short-burst low-pressure FUS exposure can be achieved under this system structure.

Our current approach can obtain FUS beam visualization/monitoring with a single short-burst FUS exposure (currently, the reconstruction can be obtained with 0.01 ms in a 1.5 MHz FUS exposure, which equates to a 10-cycle excitation). It is highly possible that the burst length can be further reduced and the SNR can been further improved. We showed that higher MI level leads to higher SNR, and introducing frame-averaging may further increase SNR (it is estimated that SNR increase was approximately∝(N − 20)^2^, where N = frame number; in dB). Yet, there are still other considerations in addition to improve SNR; for example, the acoustic pressure should not be high to induce brain tissue damage (therefore limits the exposure level increase). Second, a lower number of averaged frames should be used to guarantee sufficient real-time capability for beam pattern visualization and monitoring (therefore limits the frame-averaging number).

For *in vivo* animal FUS-BBB opening, the energy level must be sufficiently high to achieve the therapeutic effect. The level of ultrasonic energy deposition dominates the success of the therapeutic effect induction. For example, for ultrasound-induced blood-brain barrier opening, the ultrasound exposure threshold is 0.46, whereas a MI exceeding 0.8 creates safety concerns because RBC extravasations could be occurring during intervention. Thus, exposure calibration including correct position and exact energy are critical because the energy required for therapy comes with a high risk and may induce damage to the brain tissue. We showed that the reconstruction emission SI level well reflect the treatment outcomes (see Fig. 9). Increased exposure level can be well reflected in the SI level increase (a 150% exposure MI increase reflected a 160% SI increase of acoustic wave reconstruction map), and can well reflect the focal beam distortion during exposure (SI level can well reflect true energy deposition in the brain even when identical exposure level was delivered). It implies the potential of this approach in serving as valid imaging feedback for monitoring the FUS-BBB opening procedure on small animals.

In transcranial FUS-BBB opening, the skull causes ultrasound propagation energy attenuation, wave diffraction, and focal beam distortion^33^. Since the skull structure is inhomogeneous, it can be challenging to predict the FUS wave penetration. Skull-insertion effects have also been shown to degrade the reliability of transcranial passive mapping^13^,^28^, and create detection limits associated with skull thickness^34^ and density^35^. All of these effects depend on the FUS exposure frequency^34^,^35^, and should be taken into account along with nonlinearity effects^36^ and incident angle effects^37^. We also previously determined that the ultrasound exposure when transmitting through rat, swine, and human skull can have 10%, 30%, and 60% decay, respectively^38^. It might be a challenge to successfully reconstruct the backscattered acoustic wave when penetrating the human skull, particularly for the frequency range currently applied for clinical diagnostic ultrasound. A possible solution is to reduce the transmission/reception ultrasound frequency to be close or identical to the frequency applied in therapeutic ultrasound^28^, but may with the price of spatial resolution reduction.

Due to data-accessing limitation of the employed diagnostic ultrasound system, we only implemented semi real-time beam pattern visualization and monitoring with the rate of 5s/frame, and is insufficient to realize the real-time ultrasound exposure level regulation at current stage. Yet, the proposed method provide possibility to be implemented to serve as a feedback for ultrasound exposure level control during FUS intervention^39^. Future directions toward real-time implementation may include improvement of data-accessing capability of channel radio-frequency data of the imaging engine, parallel-processing and algorithm implementation in graphic processing unit (GPU), or future simplification of the implemented algorithm.

## Conclusions

In summary, we describe a synchronized backscattered ultrasound acoustic-wave reconstruction approach to visualize short-burst low-pressure FUS beam patterns in this study. We demonstrate the feasibility of using this approach to guide FUS-BBB opening via small animal experiments, and we have shown that the reconstruction of short-burst low-exposure-level ultrasonic beam is possible without the involvement of acoustic cavitation or microbubbles. Our results suggest that the proposed approach provides potential in guiding FUS brain drug delivery.

## Materials and Methods

### System implementation and experimental setup

The experiments were performed in an acrylic tank (30 cm L  × 20 cm W × 20 cm H) filled with degassed water. In order to determine the general applicability of the proposed approach, we tested four different FUS frequencies and configurations: (1, 2) a dual-confocal 0.55/1.1-MHz transducer (inner element: 1.1 MHz, active area: 45.30 mm, focal depth: 51.74 mm; outer element: 0.550 MHz, active area: 64.00 mm O.D × 46.80 I.D., focal depth: 51.74 mm), (3) a 1.5-MHZ transducer with a rectangular central opening (diameter = 64.00 mm, curvature radius = 31.64 mm, center rectangular cutout = 52.00 × 19.00 mm), and (4) a 2-MHz transducer (focal length: 55 mm, diameter = 55 mm; Sonic concepts, Woodinville, USA).

A commercialized diagnostic ultrasound imaging platform (Z.one, ZONARE Medical Systems, Inc., Mountain View, CA) was employed in this study. In order to synchronously perform the experiments between FUS exposure and diagnostic ultrasound imaging frame, the ultrasound system was set up to generate the trigger output for system synchronization, with the use of a delay generator (MODEL DG535, Digital Delay/Pulse Generator, Stanford Research Systems). The delay generator was used to externally trigger the signal generator (Agilent 33220A, USA) and then feed into a power amplifier (Model 100A250A, Amplifier Research, USA) for FUS energy exposure output. A power meter (Model 4421, BIRD, USA) was connected to monitor the FUS exposure power. During ultrasound sonication, a IQscan research package with a L10-5 linear array transducer (Bandwidth: 10–5 MHz) was used to receive IQ data signals for backscattered acoustic wave reconstruction, while the PC port was employed to send commands to the imaging system through RS-232 communications and to collect and store data for offline processing.

To acquire passive signals, diagnostic ultrasound platform was operated in receive-only mode (with the transmitter turned off), and synchronization between the receiver and the ultrasonic firing was achieved using a frame-trigger signal tapped from the ultrasound probe connector. A command script was created on a PC to automatically set up the desired system settings, and to acquire a number of frames of passive channel IQ data using a 64-element aperture. The data capture cycle was first conducted using the left-half aperture, and then repeated using the right-half aperture. The left and right half-aperture signal I/Q frames were combined to determine the effective 128-element aperture in the off-line process. Due to data-accessing limitation of the employed diagnostic ultrasound system, the time required time to reconstruct a single frame (including the data transmission and processing) was about 5 s (see the Supplementary movies).

### *In vitro* experiments

Figure S2A describes the system setup for the *in vitro* experiments, which can be divided into three parts: synchronization, FUS exposure and diagnostic ultrasound imaging. The homogeneous graphite phantoms used in the study were made of degassed water, agarose and graphite powder in certain proportion, with a size of 50 × 50 × 70 mm^3^, a sound speed of 1541 m/s at 22 °C, a density of 1040 kg/m^3^ and an attenuation coefficient of 0.45 dB/cm/MHz^40^,^41^. The phantoms were immersed in a tank filled with degassed water and carefully fixed to prevent any motion during US imaging and FUS sonication. Under the tissue-mimicking phantom, a rubber-type sound absorber was placed to reduce ultrasound wave reflection from the acrylic tank bottom, as well as the transducers with their orientation been fine-tuned to eliminate the standing waves.

The pressure levels of the FUS transducers were measured with a calibrated hydrophone (polyvinylidene-difluoride-type, HNP-0400, ONDA, Sunnyvale, CA, USA; calibrated range: 1–20 MHz). In order to verify the detection limit of the proposed reconstruction approach, a FUS sonication experiment with various exposure levels and burst lengths was performed. We used four exposure frequencies (0.55, 1.1, 1.5, and 2 MHz) with the exposure peak negative pressure ranging from 0.391–1.194 MPa (equivalent to acoustic power varying from 3 to 30 W, respectively) and the burst length ranging from 0.01 to 10-ms (tested in 1.5-MHz FUS transducer exposure).

### Magnetic Resonance Imaging (MRI)

Besides of ultrasound imaging and the reconstructed backscattered acoustic wave reconstruction mapping, MRI scans of the rat brain were also performed one day prior to *in vivo* animal experiment to serve as a high-resolution anatomical reference for backscattered ultrasound reconstructed maps. In order to align MR and diagnostic ultrasound imaging, the shaved rats’ head were attached two fiducial markers on shaved scalp throughout experimental process, which were both detectable in MRI and diagnostic imaging. It is thought that while in-prior obtained MRI provides a high-resolution anatomical reference, ultrasound imaging provides a high temporal resolution anatomical position orientation to allow coordination of the reconstructed focal beam pattern distribution. A 7-Tesla magnetic resonance scanner (Bruker ClinScan, Germany) and a 4-channel surface coil were employed. During imaging acquisition, the animals were anesthetized through inhalation of 2% isoflurane throughout the MRI process, placed in an acrylic holder and positioned at the center of the magnet. A gradient echo FLASH sequence was set to acquire animal T1-weighted images (pulse repetition time (TR)/echo time (TE) = 300/3.81 ms; FOV = 21 × 25 mm^2^; in-plane resolution = 0.25 × 0.2 mm^2^; slice thickness = 0.5 mm; flip angle = 70°).

### *In vivo* experiments

All animal procedures were approved by the Institutional Animal Care and Use Committee of Chang-Gung University and adhered to the experimental animal care guidelines. A total of 12 adult male rats (250–300 g) were exposed to high-intensity FUS-BBB sonication in the study. The experimental setup for the *in vivo* experiments was shown in Fig. S2B. A center-opened 1.5-MHz FUS transducer was used to generate concentrated ultrasound energy, with the diagnostic ultrasound probe fit-in and was confocally aligned with the focused transducer for passive backscattered emissions receiving. One arbitrary-function generator was used to produce the driving signal, which was fed to a power amplifier operating in short-period mode. Animals were anesthetized by intraperitoneal injection of chlorohydrate (30 mg/kg). The scalp of animal was shaved with clippers. PE-50 catheter was inserted into the tail vein. The animal was placed directly on an acrylic water tank (with a window of 4 × 4 cm^2^ bottom sealed with a thin film to allow ultrasound penetration), and its head attached tightly to the thin-film window with gel filling in the air gap. Diagnostic ultrasound probe was installed in the center-opened confocally aligned with the FUS transducer. For backscattered emission map reconstruction, a number of FUS exposure bursts were delivered (0.11–0.705 MPa; burst length = 10 ms) prior to microbubble administration to allow backscattered emissions signal collection. SonoVue® SF6-coated ultrasound microbubbles (2–5 μm, 30 μg/kg; Bracco Diagnostics Inc., Milan, Italy) were administered intravenously before treatment. The animal hemisphere brain site was then exposed to burst-tone mode ultrasound to locally open the BBB (peak negative pressure = 0.11–0.705 MPa measured after penetrating through the skull; burst length = 10 ms; pulse repetition frequency = 1 Hz; exposure time = 60 s). Four animal groups were performed. Group A defines to be the low-exposure (0.11 MPa) control group (n = 1). Group B and C both conducted 0.42 to 0.44 MPa exposures, but in group B (n = 3) we introduced an imperfect layer matching by not perfectly shaving the fur to purposely leave gas gap in the interface to compare the perfect layer matching of group C (n = 5). Group D introduce a high exposure level to 0.7 MPa (n = 3).

### Image signal-to-noise ratios (SNR) and statistical analyses

We analyzed the SNR in each region of interest (ROI) of the reconstructed maps to evaluate the beam detection capability, with the SNR defined as:


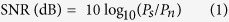


where *P*_*s*_ and *P*_*n*_ denote the signal power and noise power in the region of interest, respectively. The above quantitative data analyses were based on data acquired from the regions of interest (ROI) located in the background and the beam pattern (with the ROI dimension selected to be 1.5 mm × 1.5 mm). The unpaired Student’s t-test was used for statistically analyzing differences between groups of the SNR and peak signal intensity for both *in vitro* and *in vivo* study. Differences were recognized as statistically significant at *p* < 0.05.

## Additional Information

**How to cite this article**: Xia, J. *et al*. Low-Pressure Burst-Mode Focused Ultrasound Wave Reconstruction and Mapping for Blood-Brain Barrier Opening: A Preclinical Examination. *Sci. Rep.* **6**, 27939; doi: 10.1038/srep27939 (2016).

## Supplementary Material

Supplementary Information

Supplementary video
