# Supplementary material for: Low-Pressure Burst-Mode Focused Ultrasound Wave Reconstruction and Mapping for Blood-Brain Barrier Opening: A Preclinical Examination
Source: Sci Rep. 2016 Jun 13;6:27939. doi: 10.1038/srep27939 (PMC4904799; doi:10.1038/srep27939)
Supplement: Supplementary Information [file srep27939-s1.doc]

**Supplementary Information**

**Short-Burst Low-Pressure Focused Ultrasound Wave Reconstruction and Mapping for Blood-Brain Barrier Opening Guidance: A Preclinical Examination**

Jingjing Xia^1,2^ Po-Hsiang Tsui^1,3^, and Hao-Li Liu^1,3^

^1^Department of Electrical Engineering, Chang Gung University, Taoyuan, TAIWAN

^2^School of Electronic Information Engineering, Tianjin University, Tianjin, CHINA

^3^Medical Imaging Research Center, Institute for Radiological Research, Chang Gung University and Chang Gung Memorial Hospital at Linkou, Taoyuan, Taiwan

**SUPPLEMENTARY METHODS**

**Theory of backscattered acoustic-wave reconstruction**

FUS emissions cause interference in space and produce backscattered acoustic waves. The origin of the backscattered signals can be determined based on the precise calculation of arrival time at a piezoelectric element. To accurately reconstruct the backscattered acoustic wave from a designated target point based on the triggered ultrasound energy excitation at a specific time point, a fixed source location ideally give the same difference in arrival time at a set of receivers, and changes in the arrival time can be used to resolve different source locations. The solution requires identifying the source position of the backscattered acoustic wave based on the acoustic wave signal that is received from a plural of elements under a transmit/ receive sequence synchronization when intending to detect transmitted patterns under short and low-pressure burst exposure. A fixed source location always gives predetermined differences for time of arrival (DToA), so under perfect transmit/ receive synchronization, it is feasible to designate the corresponding DToA to obtain the backscattered acoustic waves originating from specific target positions. And since the reconstructed acoustic wave level is proportional to the ultrasound exposure energy **(when the pressure higher than the detectable threshold level)**, the mapping of the reconstructed acoustic wave theoretically represents the pressure distribution of the FUS beam. Detailed theory and derivation of the backscattered passive acoustic wave receiving and mapping are shown in the following paragraph.

Figure S1 is a schematic demonstrating how the position information is localized and reconstructed at a selected target point that is ultrasonically excited by multiple piezoelectric ultrasonic elements. Consider a plural of N elements that excite the ultrasound energy for a target position $\text{S}_{i}(x_{\text{i}},z_{i})$. The energy at the target point is the combination of energy contributions of N piezoelectric elements. Equation (1) defines the receiving ray path $R_{xn}$of a single source point in space:

$R_{xn}\text{（}x_{\text{i}},z_{i},x_{n}\text{）}=\sqrt{d_{xn}^{2}+(z_{i}+\gamma)^{2}}=\sqrt{(x_{i}-x_{n})^{2}+(z_{i}+\gamma)^{2}}$ (S1)

where $(x_{\text{i}},z_{i})$ denotes the coordinate position in the X-Z plane, $\gamma$ is the thickness of the receiving lens, $\text{x}_{\text{n}}$ denotes the position of the n-th piezoelectric ultrasonic receiving element, $n=-\text{N}/2,-\text{N}/2+1,\cdots,\text{N}/2$, N is an even number of channels, and $\text{d}_{xn}$ is the distance between $\text{x}_{i}$and $\text{x}_{\text{n}}$.

Thus, the total receiving ray path $\overline{\text{R}_{X}}$ of a single source point can be expressed as a vector of the path integration over a total of N receiving elements:

$\overline{\text{R}_{X}}\left(x_{\text{i}},z_{i},\overline{\text{X}}\left(x_{-N/2},x_{-N/2+1},\cdots,x_{N/2}\right)\right)=\bigcup_{n=-N/2}^{N/2} R_{\text{xn}}(x_{i},z_{i},x_{n})$ (S2)

where $\overline{\text{X}}$ denotes the vector of position information for each element.

To calculate the differential in reception times between elements at various positions $\text{x}_{\text{n}}$ and a reference element $\text{x}_{i}$, and taking into account the lens delays, the receiving path delay vector $\overline{T_{R_{X}}}$ of N receiving elements for a single source point can be given as equation (3):

$\begin{matrix} \overline{T_{R_{X}}} \\ =\overline{T_{R_{X}}}\left(x_{\text{i}},z_{i},\overline{\text{T}}\left(t_{-N/2},t_{-N/2+1},\cdots,t_{N/2}\right)\right) \\ =\overline{R_{\text{X}}}\times\left(\frac{1}{c_{0}}+\frac{\gamma}{c_{y}\times(z_{i}+\gamma)^{2}}\right) \\ =\bigcup_{n=-N/2}^{N/2} R_{\text{xn}}(x_{i},z_{i},x_{n})\times\left(\frac{1}{c_{0}}+\frac{\gamma}{c_{y}\times(z_{i}+\gamma)^{2}}\right) \\ =\left(\frac{1}{c_{0}}+\frac{\gamma}{c_{y}\times(z_{i}+\gamma)^{2}}\right)\times\bigcup_{n=-N/2}^{N/2} R_{\text{xn}}(x_{i},z_{i},x_{n}) \end{matrix}$ (S3)

where $\overline{\text{T}}$ is a vector composed of the receiving time $\text{t}_{n}$ from source point $\text{S}_{i}(x_{\text{i}},z_{i})$ to the N receiving elements. $c_{0}$ and $c_{y}$ are the sound speeds in the transfer medium and lens, respectively.

Then, the delay spread vector for source point goes to equation (4):

$\Delta T_{\text{i}}=\overline{T_{R_{\text{X}}}}-\left(z_{i}\times\frac{1}{c_{0}}+\gamma\times\frac{1}{c_{\gamma}}\right)$ (S4)

Accordingly, the vector for the time delay index can be calculated as follows in equation (5):

$\begin{matrix} \overline{T_{D_{index}}}_{1\times\text{N}} \\ =\left[t_{d_{-N/2}},t_{d_{-N/2}+1},\cdots,t_{d_{n}},\cdots,t_{d_{N/2}}\right]_{1\times\text{N}} \\ =\frac{f_{sRF}}{\text{c}_{0}}+\Delta T_{i}\times f_{sRF}+z_{0} \\ =\frac{f_{sRF}}{\text{c}_{0}}+\left(\overline{T_{R_{\text{X}}}}-\left(z_{i}\times\frac{1}{c_{0}}+\gamma\times\frac{1}{c_{\gamma}}\right)\right)\times f_{sRF}+TD_{\gamma}\times f_{sRF} \\ =f_{sRF}\times\left[\frac{1}{c_{0}}+\left(\overline{T_{R_{\text{X}}}}-\left(z_{i}\times\frac{1}{c_{0}}+\gamma\times\frac{1}{c_{\gamma}}\right)\right)+TD_{\gamma}\right] \\ =f_{sRF}\times\left\{\frac{1}{c_{0}}+\left[\left(\frac{1}{c_{0}}+\frac{\gamma}{c_{y}\times(z_{i}+\gamma)^{2}}\right)\times\bigcup_{n=-N/2}^{N/2} R_{\text{xn}}(x_{i},z_{i},x_{n})-\left(z_{i}\times\frac{1}{c_{0}}+\gamma\times\frac{1}{c_{\gamma}}\right)\right]+TD_{\gamma}\right\} \end{matrix}$(S5)

Where $\text{t}_{d_{\text{n}}}$ represents the various delay times between elements at various positions $\text{x}_{\text{n}}$ and the reference element $\text{x}_{i}$ with$n=-\text{N}/2,-\text{N}/2+1,\cdots,\text{N}/2$. $f_{sRF}$symbolizes the sampling frequency of the channel RF data and $\text{z}_{0}=TD_{\gamma}\times f_{sRF}$ represents the zero delay index with $TD_{\gamma}$ containing the system bulk delays and the receiving ray lens delays.

Based on the definition of a map, a vector $\text{x}_{\text{M}\times1}$ with dimension M × 1could be established to map the vector $\overline{T_{D_{index}}}_{1\times\text{N}}$ of the time delay index to yield a matrix $T_{\text{M}\times N}$ containing only element 0 and element 1, while the position of element 1 of each column was determined by the respective value of each element in vector $\overline{T_{D_{index}}}_{1\times\text{N}}$:

$T_{\text{M}\times N}=x_{M\times1}\times\overline{T_{D_{index}}}_{1\times\text{N}}$ (S6)

The passively received signals in the demodulated IQ data (i.e. $\mathop{IQ}\limits^{\text{\textasciitilde\textasciitilde}} _{\text{M}\times\text{N}}$) form in origin were first resampled to channel the RF data (i.e. $\mathop{\text{RF}}\limits^{\text{\textasciitilde\textasciitilde}} _{\text{M}\times\text{N}}$) form and then beamformed in real time by delay-and-sum methods common to clinical Bscan imaging systems. The re-modulation progress between these two matrixes was defined as equation (7):

(S7)

where $f_{mixer}$ is the re-modulation frequency. $T1_{M\times M}$ and $T2_{M\times M}$ symbolize the time vector to match the sample matrix $S_{M\times m}$. * symbolizes the conjugation. Re symbolizes the real part and Im is the imaginary part.

After taking into account the filter factor F, summing the re-modulated channel RF data over the time delay index yields the beamformed RF data (point spread function) of source point $\text{S}_{i}(x_{\text{i}},z_{i})$, which is written in equation (S8):

$\begin{matrix} \mathop{\text{rf}}\limits^{\text{\textasciitilde\textasciitilde}} (x_{i},z_{i}) \\ =F\otimes rf(x_{i},z_{i}) \\ =F\otimes\sum_{n=1}^{N} \left(\sum_{m=\overline{T_{D_{index}}}} \mathop{\text{RF}}\limits^{\text{\textasciitilde\textasciitilde}} _{\text{M}\times\text{N}}\right) \\ =F\otimes\sum_{n=1}^{N} \left(\sum_{\text{n}=1}^{N} (\mathop{\text{RF}}\limits^{\text{\textasciitilde\textasciitilde}} _{\text{M}\times\text{N}})^{T}\times T_{M\times N}\right) \\ =F\otimes\sum_{n=1}^{N} \left(\sum_{\text{n}=1}^{N} \left(\left(T1_{M\times M}\cdot\times S_{M\times m}\times\mathrm{Re}(\mathop{\text{IQ}}\limits^{\text{\textasciitilde\textasciitilde}} )_{\text{m}\times\text{N}}-T2_{M\times M}\cdot\times S_{M\times m}\times\mathrm{Im}(\mathop{\text{IQ}}\limits^{\text{\textasciitilde\textasciitilde}} )_{\text{m}\times\text{N}}\right)^{T}\times x_{M\times1}\times\overline{T_{D_{index}}}_{1\times\text{N}}\right)\right) \\ =F\otimes\sum_{n=1}^{N} \left(\sum_{\text{n}=1}^{N} (\mathop{\text{RF}}\limits^{\text{\textasciitilde\textasciitilde}} _{\text{M}\times\text{N}})^{T}\times x_{M\times1}\times\overline{T_{D_{index}}}_{1\times\text{N}}\right) \end{matrix}$ (S8)

where $\mathop{\text{rf}}\limits^{\text{\textasciitilde\textasciitilde}} (x_{i},z_{i})$ is the filtered version of $\text{rf}(x_{i},z_{i})$, is convolution.

The value of $\mathop{\text{rf}}\limits^{\text{\textasciitilde\textasciitilde}} (x_{i},z_{i})$ in equation (8) provides energy information for the target source point $\text{S}_{i}(x_{\text{i}},z_{i})$ and extension to all backscattered emission points in the imaging space.

**Histological examination**

**Albumin-bound EB dye (3% in saline) was bolus injected IV (2 mg/kg) immediately after FUS exposure experiments, and the animals were sacrificed two hours later. For sacrifice, all animals were first deeply anesthetized with 10% chloral hydrate and infused with heparinized saline through the cardiac ventricle until colorless infusion fluid was obtained from the atrium. After animal sacrifice, the brain was serially sectioned (2-μm thickness) with the slicing orientation identical to imaging direction. Representative sections were stained with hematoxylin and eosin (HE) in order to characterize erythrocyte extravasations.**

**SUPPLEMENTARY FIGURES**


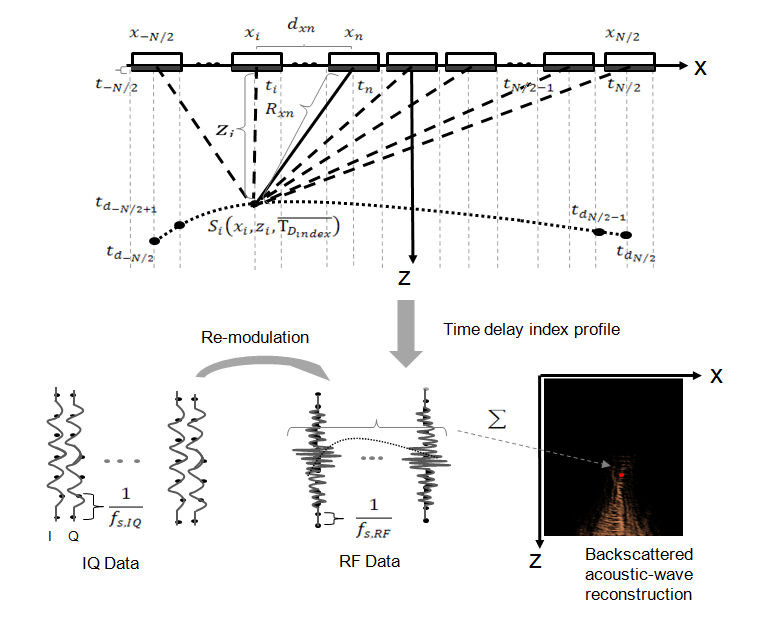


**Fig. S1.** Theoretical diagrams of the backscattered acoustic mapping reconstruction. The diagram shows a schematic showing position information localization and reconstruction at a selected target point which is ultrasonic excited from a plural of piezoelectric ultrasonic elements


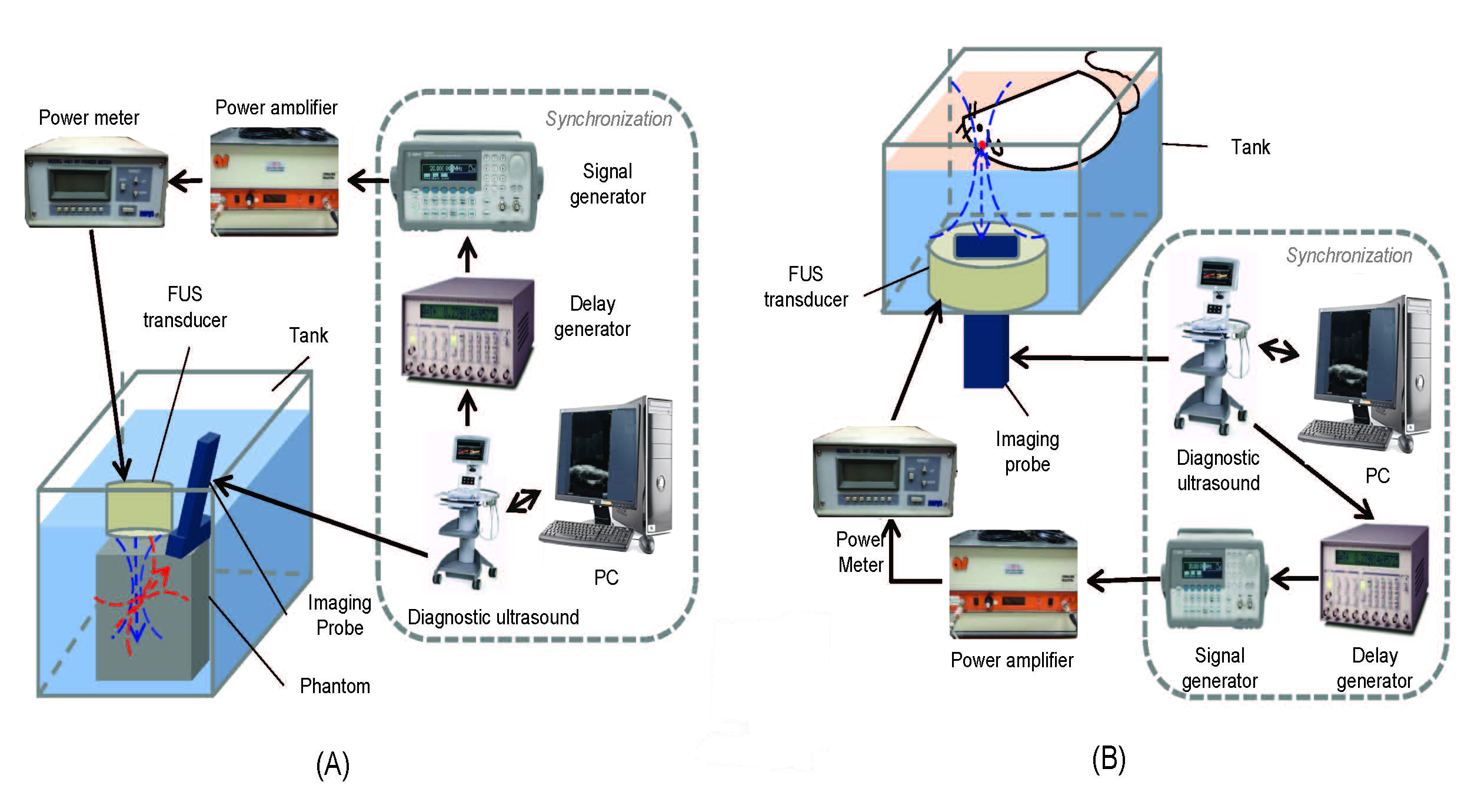


**Fig. S2.** Experimental setup. (A) *in vitro* experiments; (B) *in vivo* experiments.


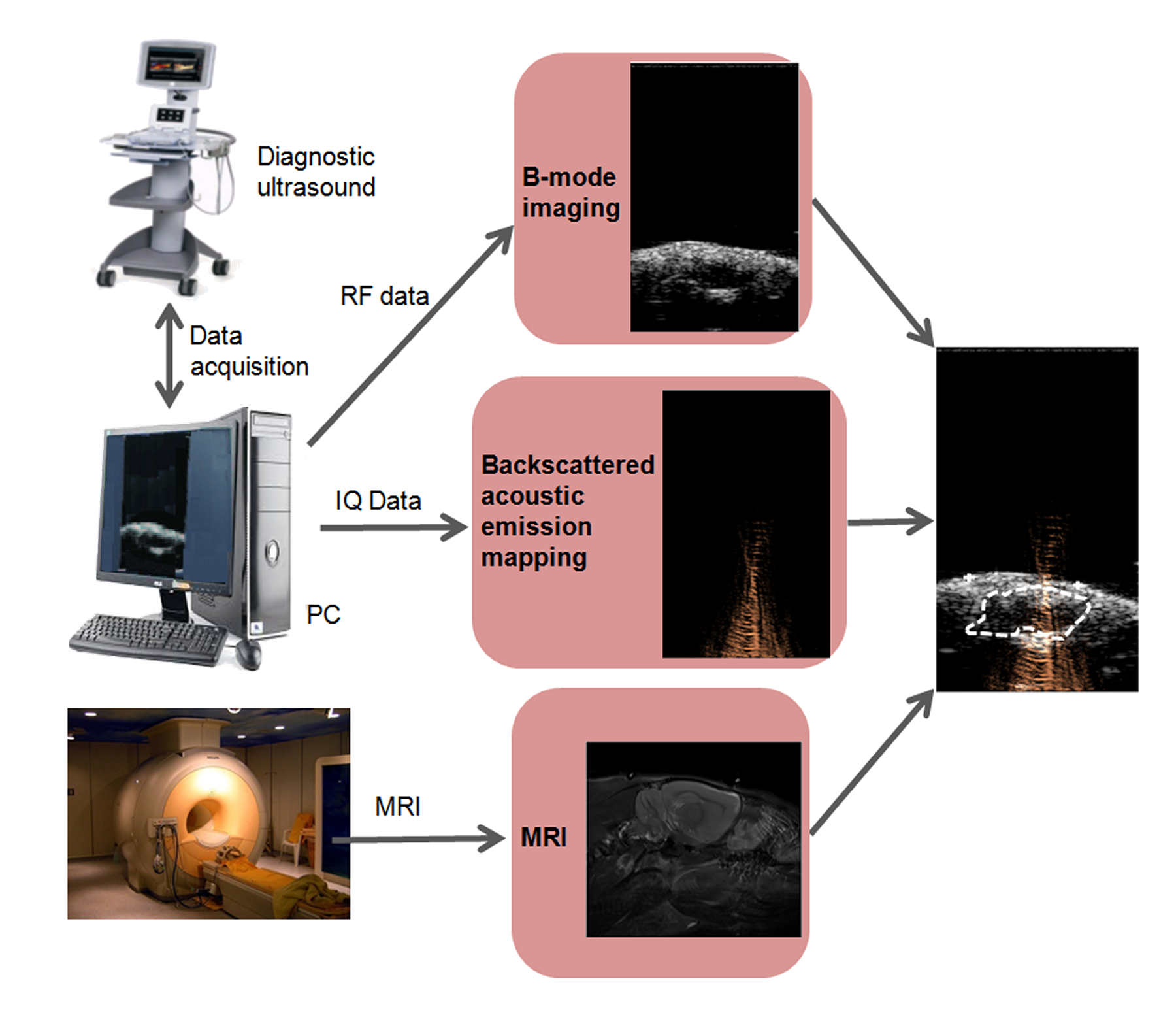


**Fig. S3.** Concepts of the proposed scheme in guiding FUS-induced BBB opening. The concept includes the co-localization of ultrasound B-mode imaging, acoustic emission reconstruction map infusion, and a prior MRI for anatomical information to fulfill focused ultrasound blood-brain barrier (BBB) opening guidance.


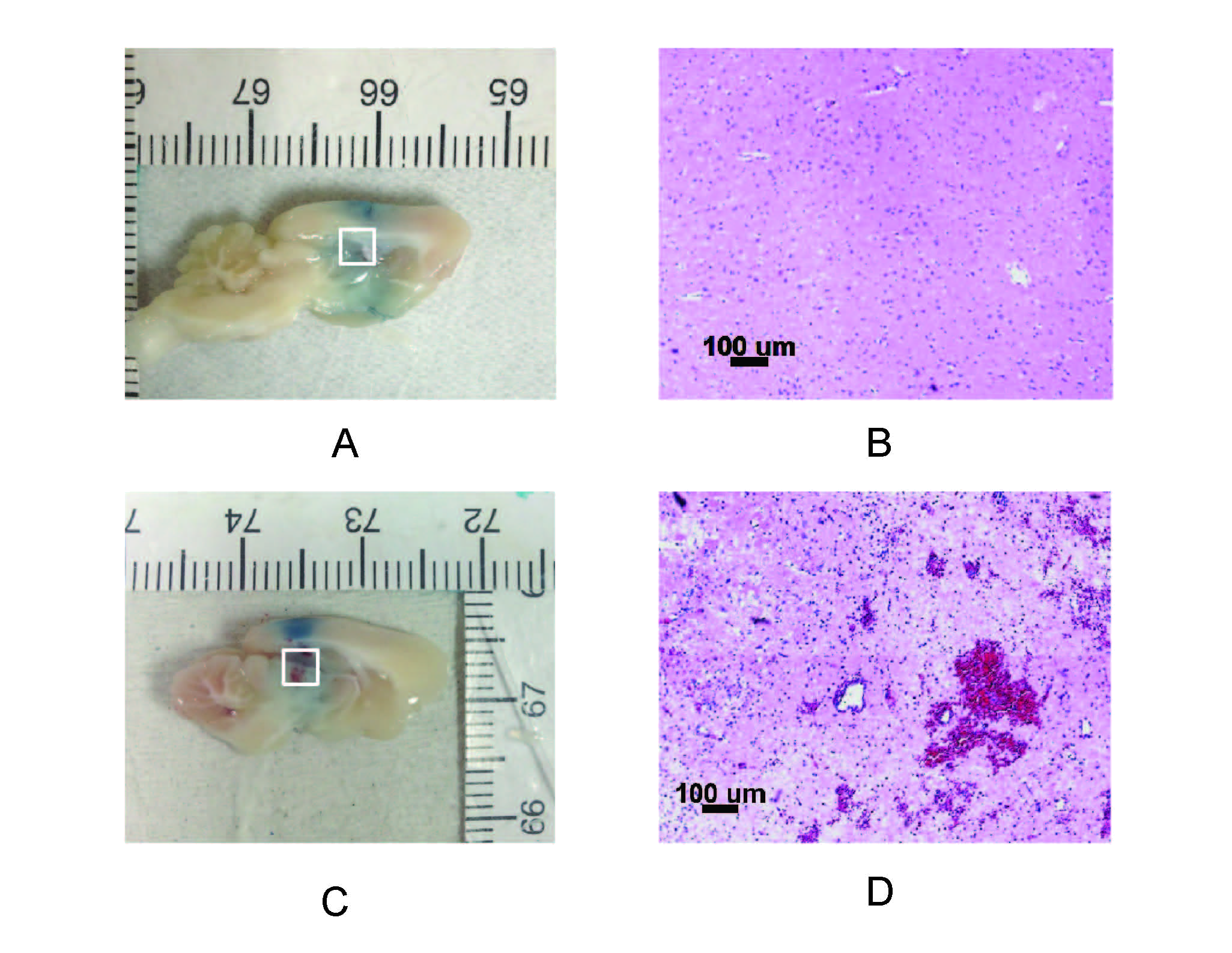


**Fig. S4. EB-stained brain sections and corresponding HE staining microscopy. (A, B) FUS exposure level of 0.44 MPa (obtained from group C); (C, D) FUS exposure level of 0.7 MPa (obtained from group D). HE stained microscopic photos (in B and D) were obtained from brain locations marked by white squares (in A and C).**

**SUPPLEMENTARY VIDEO**

**
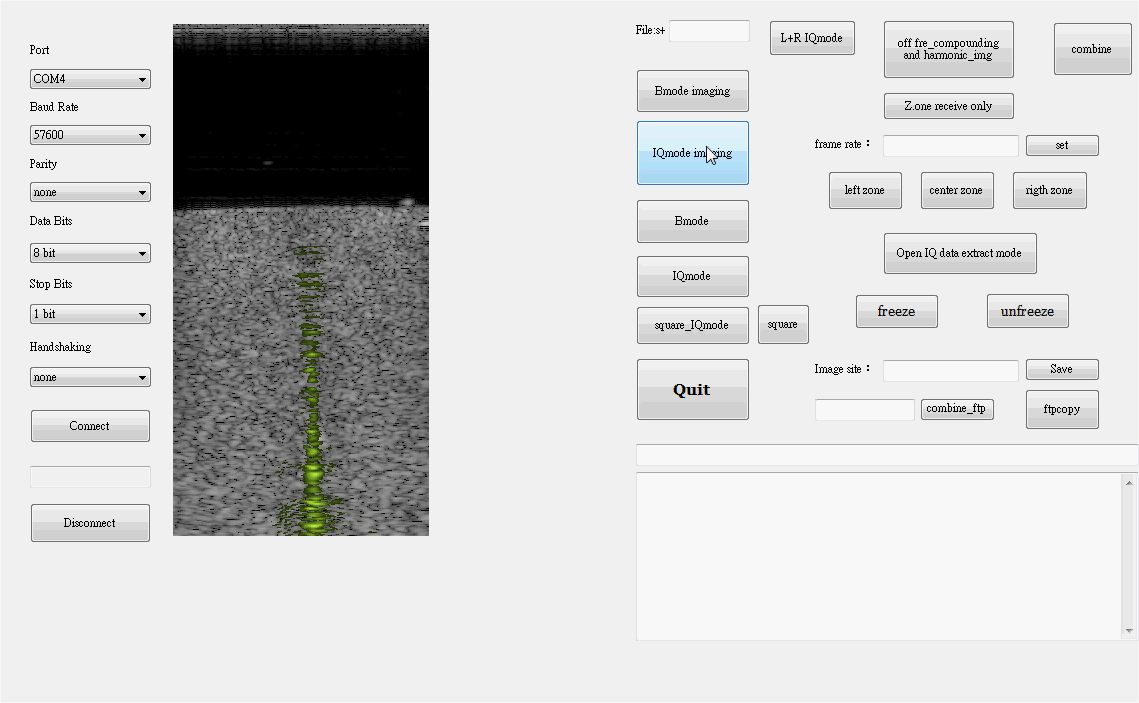
**

**Video S1.** Example showing the graphic-user-interface implementation to perform semi real-time backscattered emission reconstruction to visualize FUS pattern deposition.
